# Supplementary material for: Structural basis for assembly of the CBF3 kinetochore complex
Source: EMBO J. 2017 Dec 6;37(2):269–81. doi: 10.15252/embj.201798134 (PMC5771398; doi:10.15252/embj.201798134)
Supplement: Supplementary file 1 — Appendix [file EMBJ-37-269-s001.pdf]

APPENDIX

Structural Basis for Assembly of the CBF3 Kinetochore Complex

Vera Leber, Andrea Nans, Martin R Singleton

Appendix Figures S1-S4

Appendix Figure Legends S1-S4

Appendix Tables S1-S3

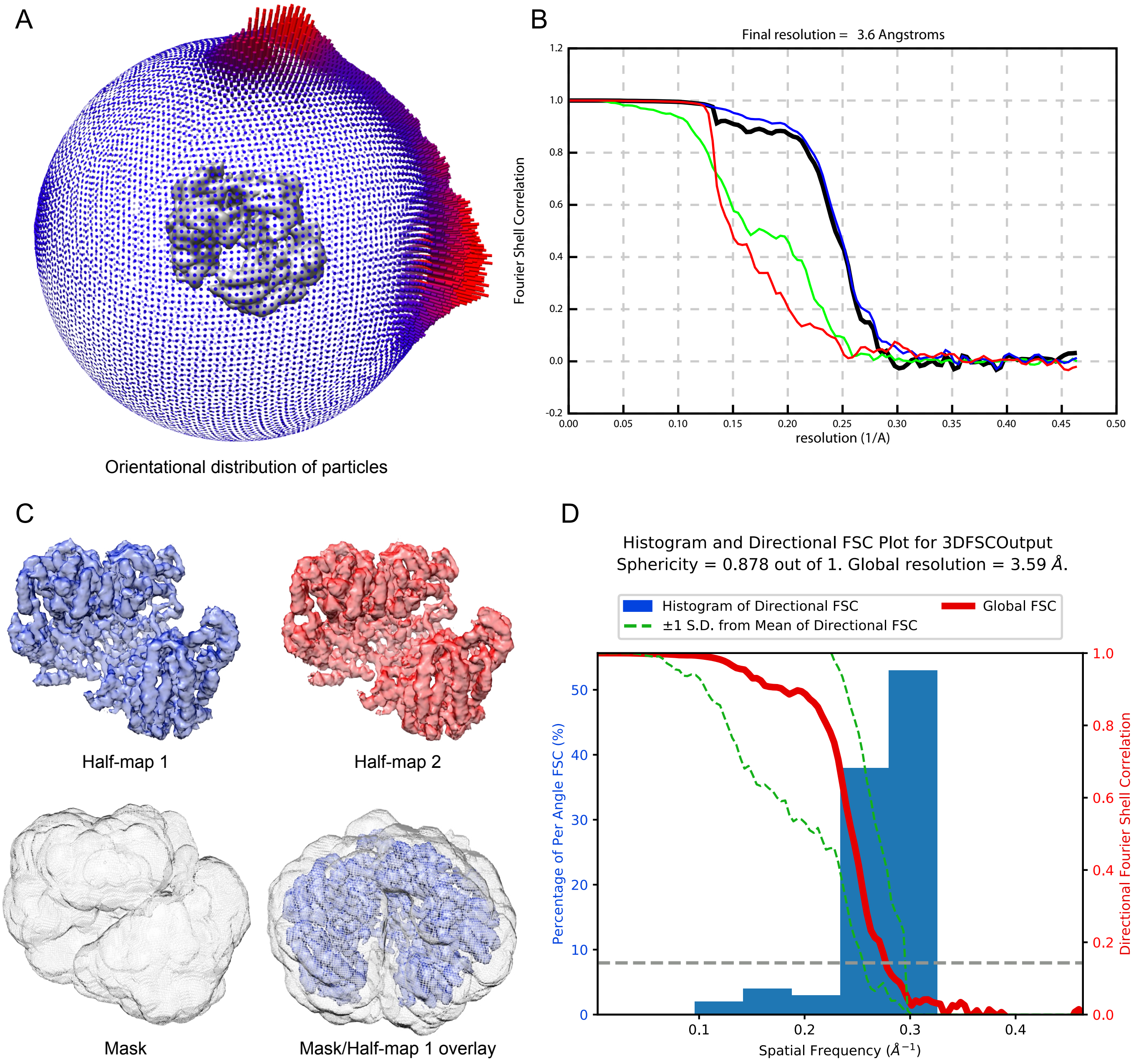

Leber et al., Appendix Figure S1

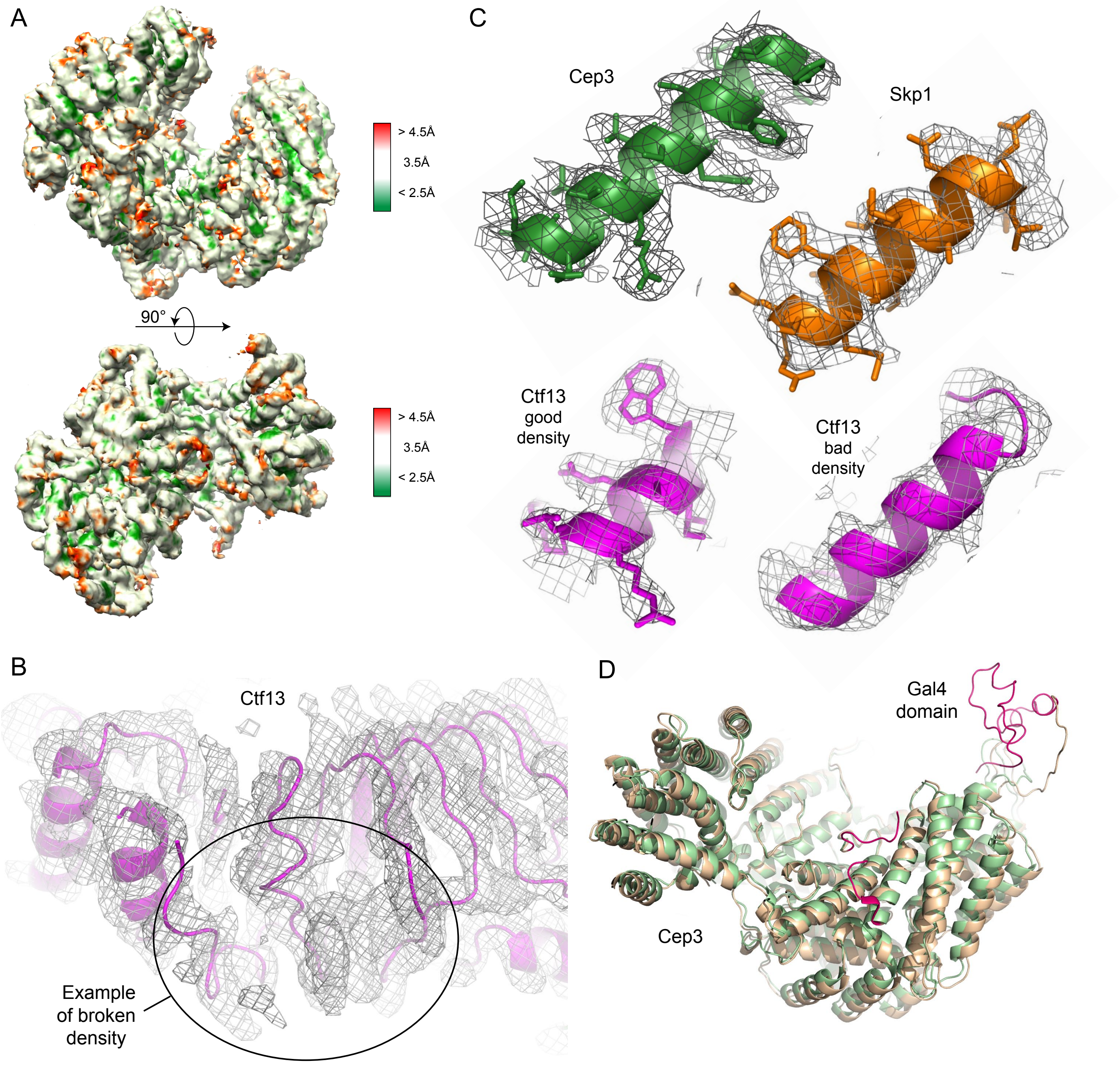

Leber et al., Appendix Figure S2

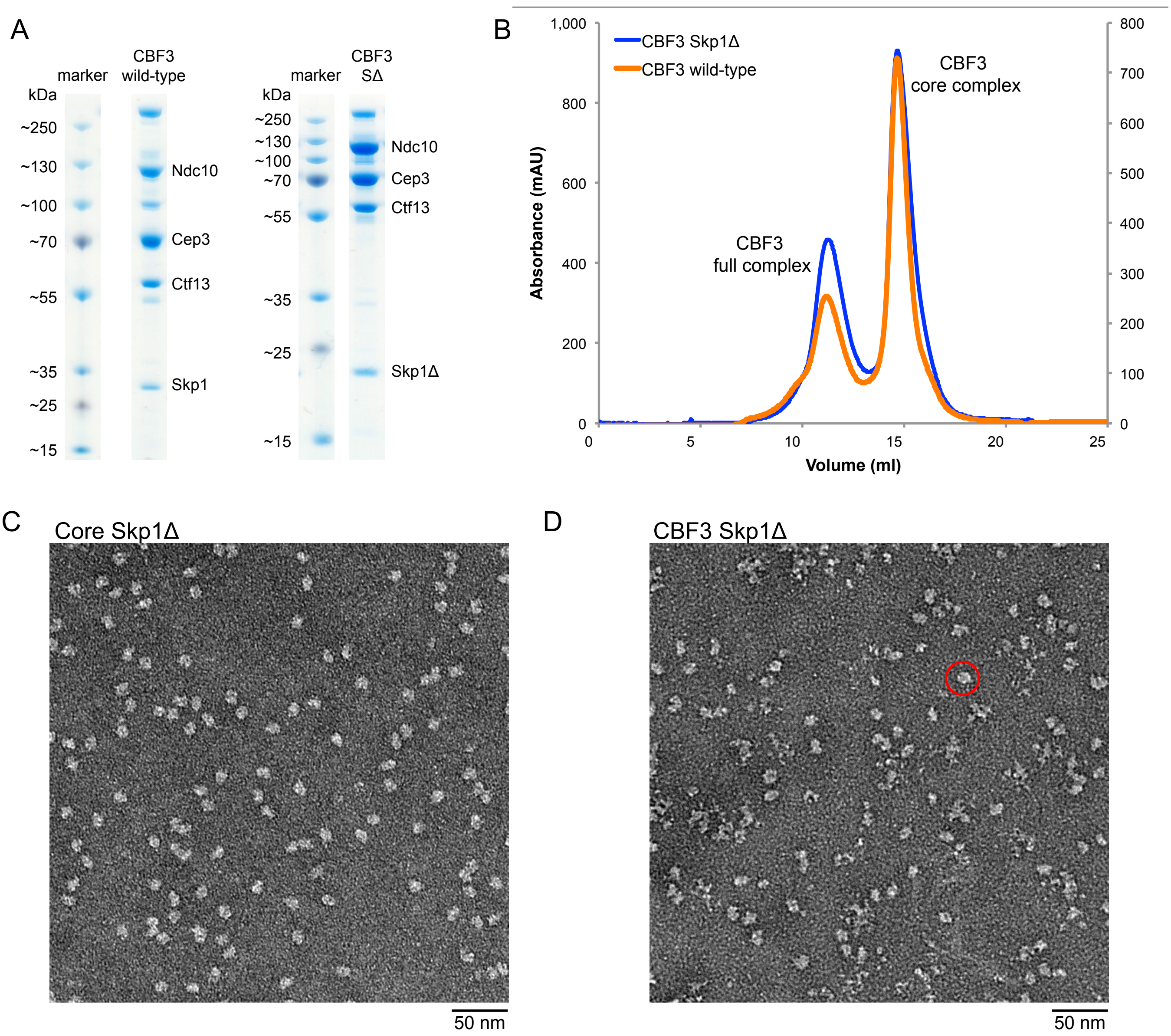

Leber et al., Appendix Figure S3

A

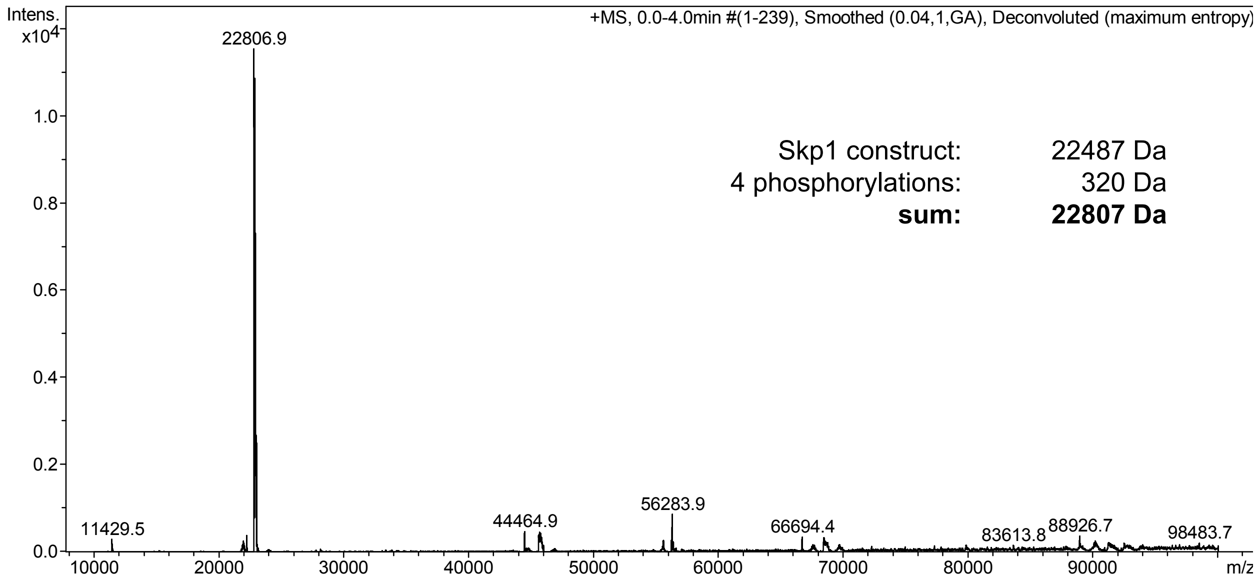

Leber et al., Appendix Figure S4

Appendix Figure S1. Further image processing data

A) Orientational distribution showing two preferred orientations, but also a good coverage of all other orientations. B) Fourier shell correlation (FSC) curve, indicating overall resolution limit of 3.6 Å. Colour codes are as following: black - corrected; blue - masked; green – unmasked and red – phase randomised. C) Both half-maps and mask used for refinement, as well as an overlay of half-map 1 and the mask. D) Assessment of resolution anisotropy in final reconstructions.

Appendix Figure S2. Local resolution and quality of cryo-EM density

A) Cryo-EM density map in two orientations and coloured according to local resolution. B) Cryo-EM density showing the broken density (black circle) of some of the LRRs of Ctf13 (shown as pink ribbon diagram). C) Example helices with corresponding EM density (shown as mesh) of Cep3 (green), Skp1 (orange) and Ctf13 (pink). For Ctf13 two helices were chosen, one in an area of good and one in an area of bad electron density. D) Differences in the Cep3 crystal structure (green) and the refined cryo-EM Cep3 structure (brown). The previously unresolved loop, as well as the previously unresolved Gal4-domain, is highlighted in red.

Appendix Figure S3. Purification and analysis of CBF3 Skp1Δ

A) SDS-PAGE/Coomassie stain of the eluate after StrepTactin purification, comparing CBF3 wild-type (left panel, 4-12% polyacrylamide-SDS gel) with CBF3 Skp1Δ (right panel; 10% polyacrylamide-SDS gel), showing that co-expression of CBF3 Skp1Δ yields more Ndc10 than wild-type CBF3. B) Overlay of the size-exclusion chromatography absorbance profiles of wild-type (orange) and Skp1Δ (blue) CBF3 core and full complex. C-D) Negative stain analysis of CBF3 Skp1Δ core (C) and full complex (D). As with wild-type CBF3, core complex particles are nicely distributed and distinct, whereas full complex is comprised of core particles (red circle) and additional diffuse density.

Appendix Figure S4. Intact mass analysis of Skp1

Mass spectrum of intact mass analysis of Skp1, showing a peak at 22806.9 Da, corresponding exactly to the Skp1 construct used plus four phosphorylations.

Appendix Table S1. Data collection and processing

Microscope	Titan Krios (FEI)
Camera	K2 Summit (Gatan)
Voltage (keV)	300
Defocus range (µm)	-1.5 to -3.5
Magnification	130,000 x
Pixel size (Å/pixel)	1.078
Dose rate (e^-/pixel/s)	6.3
Movies	8755
Frames per movie	25
Total exposure time (sec)	10
Total accumulated dose ($e^-/Å^2$)	54
Particles after autopicking	~3.5 million
Particles contributing to final reconstruction	209,751
Sharpening B-factor ($Å^2$)	-150
Final resolution (Å)	3.6

Appendix Table S2. Refinement statistics

	**Cep3**	**Skp1**
CC_Mask	0.77	0.78
rmsd (bonds, Å)	0.01	0.01
rmsd (angles, °)	0.90	0.92
Rama favoured	94.3	91.9
Rama allowed	5.7	8.1
Rama outlier	0	0
Clash score	3.33	3.5
Molprobity	1.51	1.63

Appendix Table S3: Sequences of DNA fragments used for EMSA studies

**Name**	**Sequence**
56bp WT CEN3	5′-TATTAGTGTATTTGATTTCCGAAAGTTAAAAAAGAAATAGTAAGAAATATATATTT-3′
33bp wild-type CEN3	5′-AAATATTAGTGTATTTGATTTCCGAAAGTTAAA-3′
33bp CCG mutant CEN3	5′-AAATATTAGTGTATTTGATTT*GAT*AAAGTTAAA-3′
33bp TGT mutant CEN3	5′-AAATATTAG*AAC*ATTTGATTTCCGAAAGTTAAA-3′
33bp double mutant CEN3	5′-AAATATTAG*AAC*ATTTGATTTGATAAAGTTAAA-3′
