# Supplementary material for: Structural basis for assembly of the CBF3 kinetochore complex
Source: EMBO J. 2017 Dec 6;37(2):269–81. doi: 10.15252/embj.201798134 (PMC5771398; doi:10.15252/embj.201798134)
Supplement: Supplementary file 2 — Expanded View Figures PDF [file EMBJ-37-269-s002.pdf]

Expanded View Figures

Figure EV1. Negative stain analysis and purification of core and different Ndc10 constructs.

A, B Negative stain analysis of CBF3 core (A) and CBF3 full complex (B), showing nice dispersed particles for CBF3 core, but suboptimal particles for the full complex. The latter is comprised of core particles (red circle) and additional diffuse density, assumed to be unstructured Ndc10.

C SDS–PAGE/Coomassie stain showing the initial StrepTactin pull-down of co-expressed CBF3 core and NTD and CTD of Ndc10, showing that only the NTD can be pulled out by the tagged core but not the CTD.

D Suboptimal size-exclusion profile of CBF3 core and Ndc10 NTD indicates that they do not form a stable complex.

E Negative stain micrograph of CBF3 core and Ndc10 NTD. Particles are heterogeneous and mostly resemble the core complex (circled).

 © 2017 The Francis Crick Institute

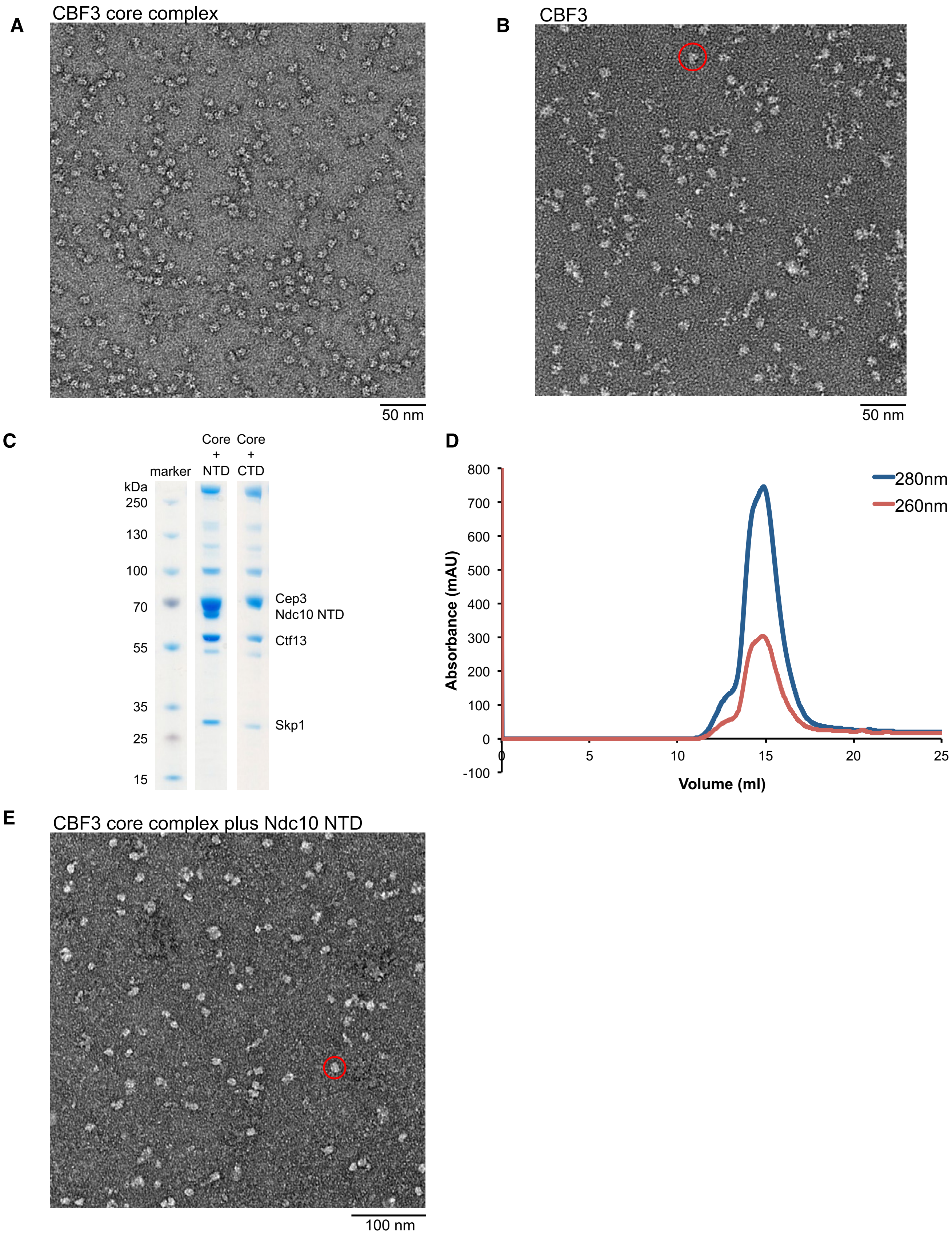
A
CBF3 core complex
50 nm
B
CBF3
50 nm
C
marker
Core + NTD
Core + CTD
kDa
250
130
100
70
55
35
25
15
Cep3
Ndc10 NTD
Ctf13
Skp1
D
280nm
260nm
Absorbance (mAU)
800
700
600
500
400
300
200
100
0
-100
0
5
10
15
20
25
Volume (ml)
E
CBF3 core complex plus Ndc10 NTD
100 nm

Figure EV1.

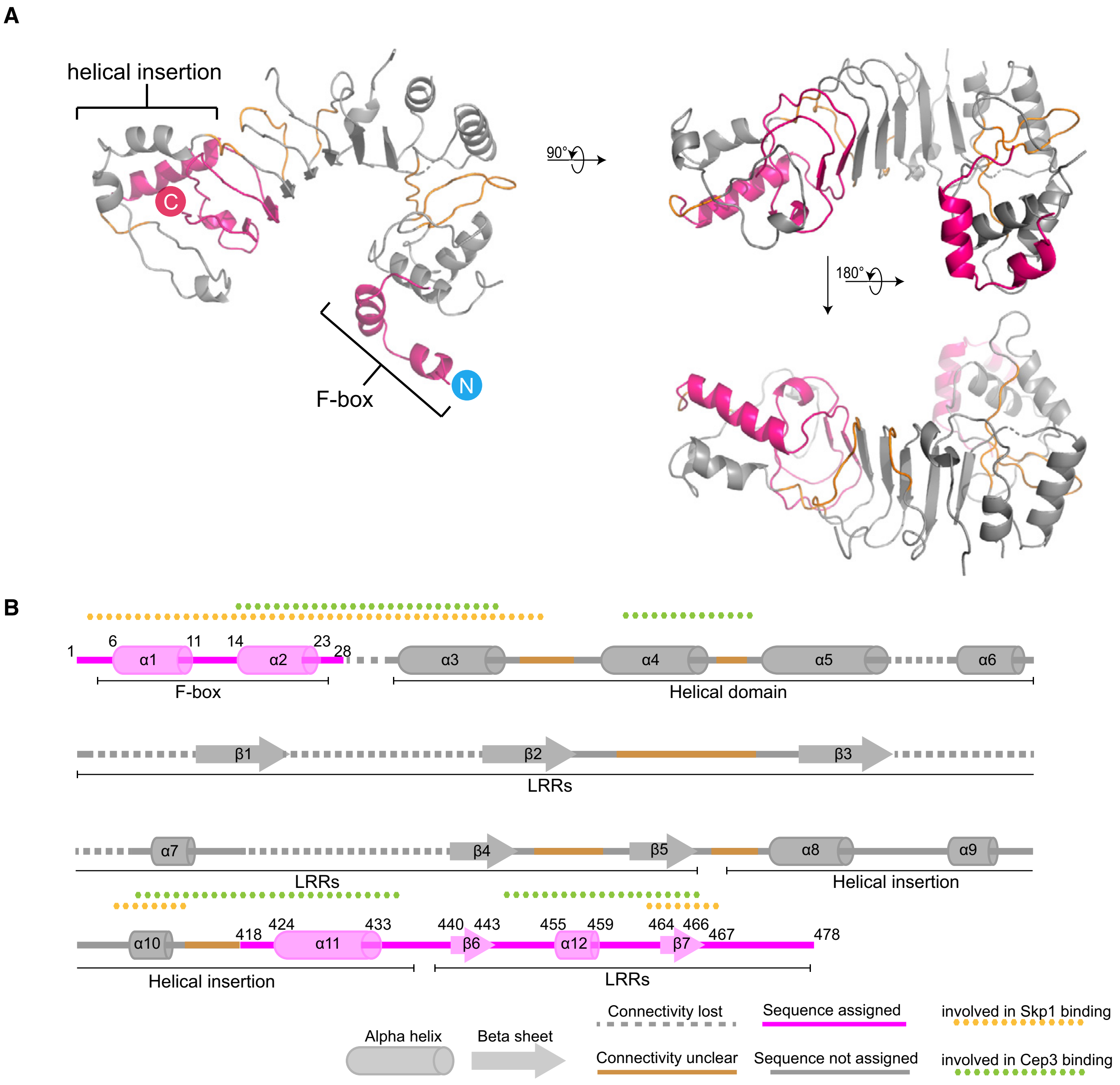

Figure EV2. Structure of Ctf13.

A Ribbon diagram of the Ctf13 structure. The location of the F-box and helical insertion are indicated. Sections depicted in pink represent parts of the structure where sequence may be reliably assigned. Sections in grey have no sequence assigned. Sections coloured in orange represent areas where the connectivity is ambiguous, but a "best-guess" may be made. Areas of poor density were not modelled; this is particularly apparent on the outer (convex) surface of the LRRs.

B Schematic linear diagram of Ctf13 providing an overview of domains and approximate location of secondary structure elements. Colour codes are as in (A). Elements involved in interaction with the other two CBF3 core subunits are also highlighted.

 © 2017 The Francis Crick Institute

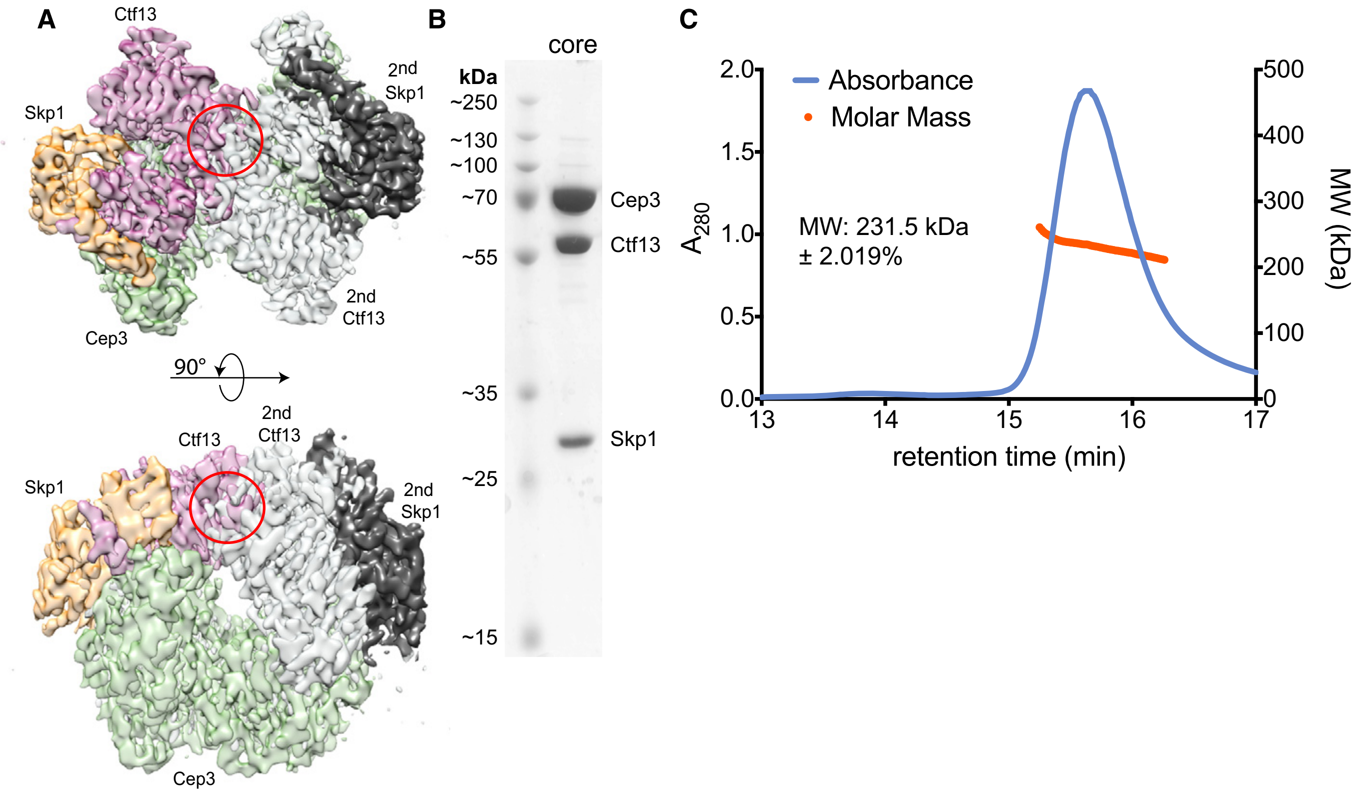

Figure EV3. Structural basis for CBF3 core stoichiometry.

A An overlay of a hypothetical second Ctf13/Skp1 heterodimer bound to the "free" Cep3 monomer, showing a steric clash between the two Ctf13 densities (red circle). The subunits are colour coded: Cep3 homodimer—green; Ctf13—pink; Skp1—orange; hypothetical second Ctf13—grey; hypothetical second Skp1—black.

B Typical SDS–PAGE/Coomassie stain of core complex after size-exclusion chromatography, showing a stoichiometry of Cep3(2):Ctf13(1):Skp1(1).

C SEC-MALS analysis of the core complex.

© 2017 The Francis Crick Institute

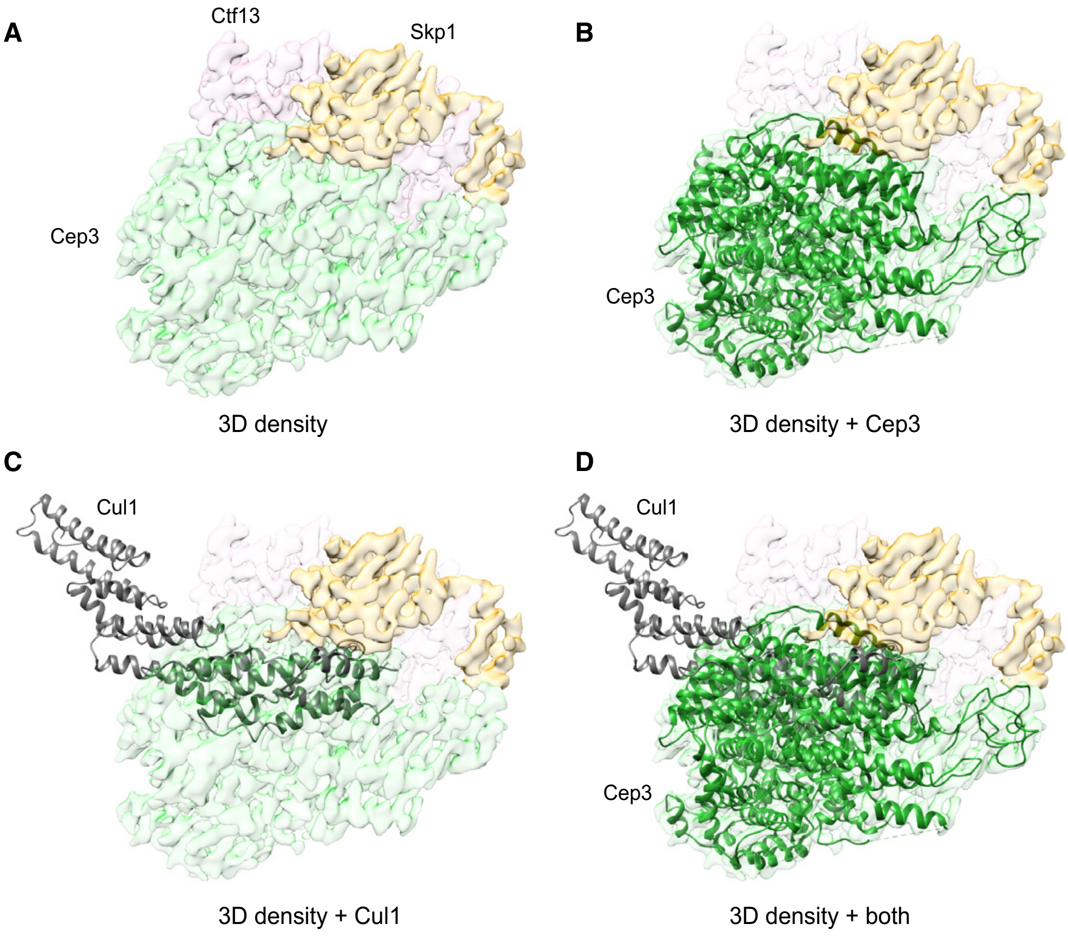

Figure EV4. Similarities between Skp1-Cep3 and Skp1-Cul1 binding interface.

A Cryo-EM density with colour-coded subunits.
B Cryo-EM density with fitted Cep3 shown as ribbon diagram (green).
C Cryo-EM density with overlaid Cul1 (PDB ID: 1ldk) shown as ribbon diagram (grey).
D Cryo-EM density with both, fitted Cep3 and overlaid Cul1, showing that they share the same interface to Skp1.

 © 2017 The Francis Crick Institute

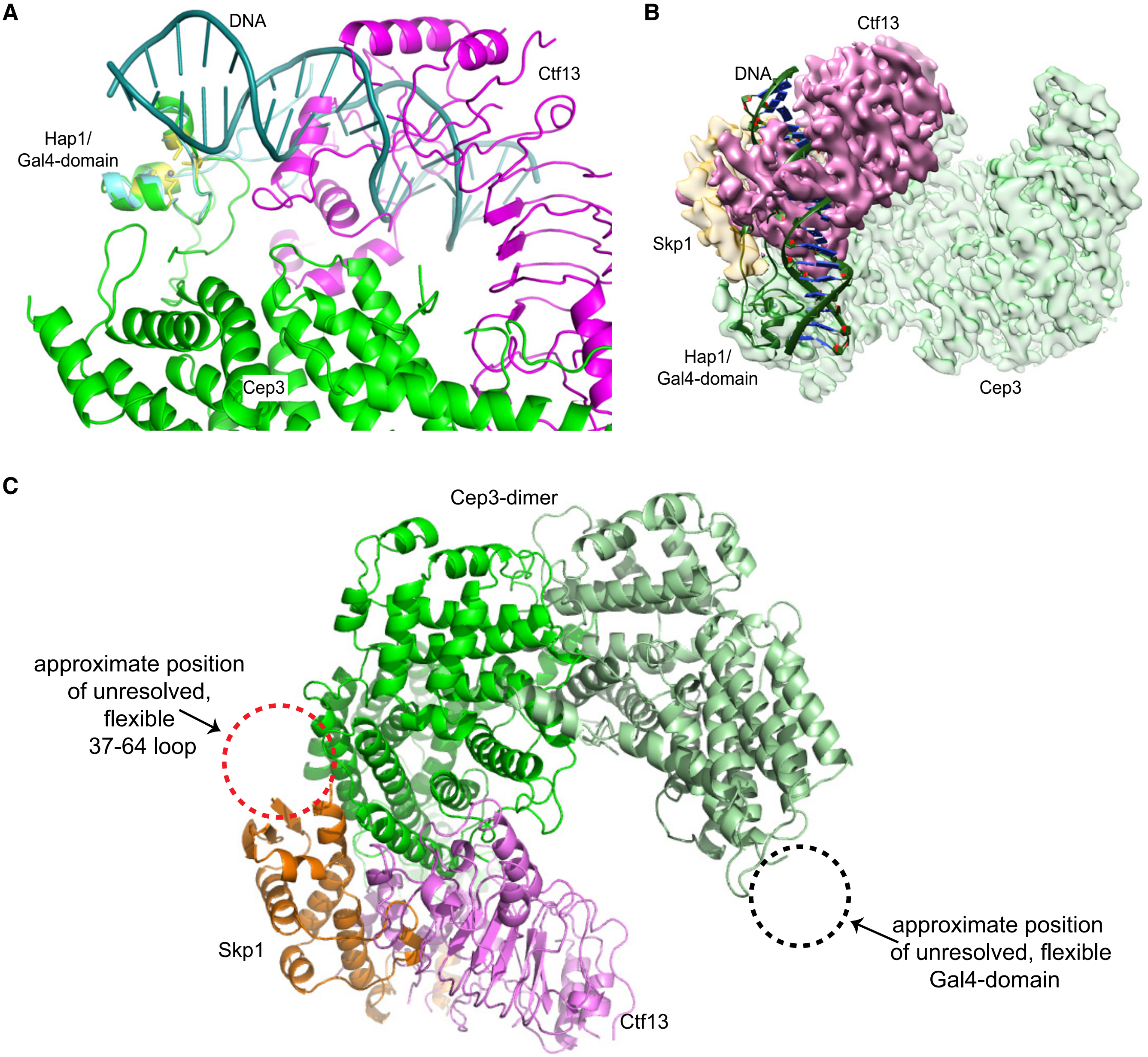

Figure EV5. Structural insight into DNA binding.

A, B An overlay of the DNA-binding domain of Hap1 bound to DNA showing a steric clash between the DNA and Ctf13. (A) Detailed view of the steric clash. (B) View of the whole cryo-EM density showing the steric clash.

C Structure of the core complex, demonstrating the likely position of the unresolved, flexible 37–64 Skp1 loop (red dotted circle) in relation to the rest of the structure, as well as the position of the second free Gal4-domain (black dotted circle).

© 2017 The Francis Crick Institute
